# Risk Assessment of Exposure to Lead in Tap Water among Residents of Seri Kembangan, Selangor State, Malaysia

**DOI:** 10.5539/gjhs.v5n2p1

**Published:** 2012-11-22

**Authors:** Lim C. S., Shaharuddin M. S., Sam W. Y.

**Affiliations:** 1 Department of Environmental and Occupational Health, Faculty of Medicine and Health Sciences, Universiti Putra Malaysia, Serdang, Selangor, Malaysia

**Keywords:** lead, first-flushed water, fully-flushed water, non-carcinogenic risk, Seri Kembangan

## Abstract

**Introduction:**

A cross sectional study was conducted to estimate risk of exposure to lead via tap water ingestion pathway for the population of Seri Kembangan (SK).

**Methodology:**

By using purposive sampling method, 100 respondents who fulfilled the inclusive criteria were selected from different housing areas of SK based on geographical population distribution. Residents with filtration systems installed were excluded from the study. Questionnaires were administered to determine water consumption-related information and demographics. Two water samples (first-flushed and fully-flushed samples) were collected from kitchen tap of each household using HDPE bottles. A total of 200 water samples were collected and lead concentrations were determined using a Graphite Furnace Atomic Absorption Spectrophotometer (GFAAS).

**Results:**

Mean lead concentration in first-flushed samples was 3.041± SD 6.967µg/L and 1.064± SD 1.103µg/L for fully-flushed samples. Of the first-flushed samples, four (4) had exceeded the National Drinking Water Quality Standard (NDWQS) lead limit value of 10µg/L while none of the fully-flushed samples had lead concentration exceeded the limit. There was a significant difference between first-flushed samples and fully-flushed samples and flushing had elicited a significant change in lead concentration in the water (Z = -5.880, p<0.05). It was also found that lead concentration in both first-flushed and fully flushed samples was not significantly different across nine (9) areas of Seri Kembangan (p>0.05). Serdang Jaya was found to have the highest lead concentration in first-flushed water (mean= 10.44± SD 17.83µg/L) while Taman Universiti Indah had the highest lead concentration in fully-flushed water (mean=1.45± SD 1.83µg/L). Exposure assessment found that the mean chronic daily intake (CDI) was 0.028± SD 0.034µgday^-1^kg^-1^. None of the hazard quotient (HQ) value was found to be greater than 1.

**Conclusion:**

The overall quality of water supply in SK was satisfactory because most of the parameters tested in this study were within the range of permissible limit and only a few samples had exceeded the standard values for lead and pH. Non-carcinogenic risk attributed to ingestion of lead in SK tap water was found to be negligible.

## 1. Introduction

Metals could exert effects that are beneficial or harmful to our human body (Caussy et al., 2003). Heavy metals are especially renowned for their toxicity effects towards human beings, aquatic life and the environment. Lead is one of the heavy metals which have no known physiologically relevant role in the body (White et al., 2007). Lead from environmental pollution is not carcinogenic, but even low dose lead exposure has been shown to have detrimental and long-lasting effects on the renal, hemopoietic and nervous system (Fertmann et al., 2004).

The main target for lead toxicity is the nervous system, both in adults and children (ATSDR, 1998). It can create irreversible intellectual impairment in infants and young children, even at blood lead levels below10 mg/dL (Lanphear et al., 2000; Gump et al., 2008; Jusko et al., 2008). As exposure continues, the effect progresses with insomnia, confusion, impaired concentration, and memory problems (Robson, 2003).

Humans are continuously exposed to lead from natural as well as anthropogenic sources (Christensen, 1995). Since the introduction of unleaded petrol and total phased out of leaded petrol in 1998, the lead level in the atmosphere had declined significantly (DOE, 2011). In addition to exposure to lead in the air, ingestion of lead in drinking water has become one of the major sources of human exposures to lead (Matte et al., 2000). The presence of lead in drinking water is a public health problem due to their absorption and possible accumulation in organisms (Chiron et al., 2003).

Drinking water in the house can be contaminated by corrosion of the plumbing materials used to supply the houses (Gulson et al., 1994). Corrosion of household plumbing systems is an important source of lead found in tap waters (Calderon, 2000). Significant levels of trace metals may be detected after stagnation of the water in distribution systems, especially during night-time (Seifert et al., 2000).

In Malaysia, the water utilities are managed and operated by both state authority and concession companies following the privatization exercise in 1987 (Lee, 2004). The water supplied to consumers has actually been through treatment stages before being distributed. The treated water that is already safe for drinking will then be pumped to the balancing reservoirs before being distributed to service reservoirs (Konsortium Abass Sdn Bhd, 2010). Although the water is tested prior to supplying to the consumers, there might be some possibility of heavy metal contamination into the water supply during transfer and storage before going to households. Comparison of the water quality determined with available standards would be another interesting part of the study.

Lead in drinking water is a major public health concern. Risk from exposure to lead contaminated water will vary, depending on the individual, the circumstances, and the amount of water consumed. For example, infants who drink formula prepared with lead-contaminated water may be at a higher risk because of the large volume of water they consume relative to their body size (CDC, 2002). If there is good reason to believe that a person may have a significant source or combination of sources of lead exposure, bio-monitoring may be prudent (Payne, 2008). Exposure and associated health risk levels of exposure to lead in tap water of the SK population has not yet been investigated.

## 2. Area Descriptions

This cross sectional study was conducted to estimate the risk of exposure to lead via tap water ingestion pathway for the Seri Kembangan (SK) population. SK was selected as the study location because of its large population density with a total population of 66,481 people and there are many housing areas including Taman Sri Serdang, Taman Universiti Indah and Taman Sungai Besi Indah (Department of Statistics Malaysia, 2000). A study conducted by Abdullah (2008) found that the Semenyih Dam, which supplies raw water to be used in areas including Seri Kembangan, was actually contaminated with heavy metals such as lead, cadmium and copper. No similar studies have been conducted in SK as yet. The list of housing areas studied can be seen in Table 1.

**Table 1:** Geographical population distribution and required water samples (N=100)

**Housing area**	**Population**	**Water samples**
Taman Serdang Raya	13120	8
Taman Serdang Jaya	18190	12
Taman Sri Serdang	14360	9
Taman Universiti Indah	14330	9
Taman Sungai Besi Indah	8295	5
Taman Belimbing Indah	3430	2
Taman Muhibah	3640	2
Kampung Baru Seri Kembangan	10175	6
Taman Bukit Serdang	70960	45
**TOTAL**	**156500**	**100**

Source: Local Planning of Subang Jaya Municipal Council (2020).

## 3. Methodology

The study population was the SK population, and the sampling unit was a resident living in SK who fulfilled the inclusion criteria, which were adult Malaysians (≥ 18 years old) who use the tap water for drinking and cooking. The exclusive criteria were tap water which is not from municipal water supply and personal water filtration systems which are installed at the house.

The sample size of this study was 100 respondents. It was calculated based on a formula by Kirkwood (2003) and taken into consideration missing and damaged data. The sampling method in this study was purposive sampling method, where the respondents were selected based on inclusive and exclusive criteria stated. The number of samples to be collected from each area of SK was calculated according to the geographical population distribution as shown in Table 1. Data collection was carried out from 21st December 2010 until 9th February 2011.

### 3.1 Study Instrumentation and Data Collection

For each sampling unit, one person who fulfilled the inclusive criteria was invited to be the respondent. The questionnaire, which inquired about socio-demographics of occupants, health status, water supply and plumbing system in the house, was administered during the visit. The questionnaires were modified from the Baseline, Descriptive and Time-Activity Questionnaires used in NHEXAS-Arizona study (Lebowitz et al. 1995) and also from a study conducted by Kavcar (2006). The questionnaire was in Malay since *Bahasa Malaysia* is our national language and the language is understood by all the citizens.

In this study, 250 ml high-density polyethylene (HDPE) bottles were used for sampling of tap water. HDPE bottles had previously been used in many studies to detect heavy metals in water (Shuhaimi-Othman et al., 2008, Kavcar et al., 2009; Cidu et al., 2010). Before sampling, the bottles were acid washed where they were soaked overnight in 10% acid nitric bath before being washed twice with distilled water. After drying in the oven, the bottles were tightly capped and sealed in plastic bags before transported to the site to avoid contamination from heavy metals the external environment.

Two water samples were collected from each house. The first water sample (first-flush sample) was the very first drops of cold water that came out from the kitchen tap after an overnight of stagnation. This sample determines whether lead accumulated in the water comes from the house's plumbing system. The second water sample (fully-flushed sample) was the water taken from the kitchen tap after flushing for 2 minutes. This sample determines if the main distribution system was the source of lead in the water.

**Figure 1:**
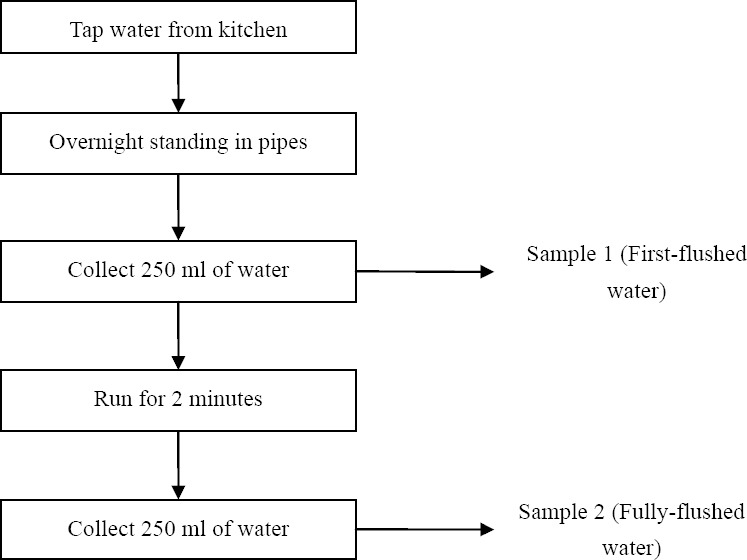
Flow diagram of sampling technique

Flow diagram of sampling technique is shown in Figure 1. Written consent was obtained from the respondent, who was given a written step-by-step instruction for collecting the water samples. The procedure followed method used by Vegesna & McAnally (1995) and Rajaratnam et al. (2002). All HDPE bottles were collected within 6 hours of sampling.

The 250ml beakers were used to fill the fully flushed tap water for testing of conductivity, TDS, temperature and pH. These parameters were measured directly *in situ* during collection of HDPE bottles from respondents (Angerville et al., 2007). Conductivity, total dissolved solid (TDS) and temperature were measured simultaneously using a HACH SensionTM5 conductivity meter while pH was measured using a LAMOTTE Tracer ORP Pocketester.

The measurement of lead content in water samples were performed using a Perkin Elmer AAnalyst 600 Graphite Furnace Atomic Absorption Spectrophotometer (GFAAS) equipped with the intuitive WinLab32 software, which featured the tools to analyze samples, report and archive data (Sarojam, 2011).

### 3.2 Quality Control

The analysis of water samples using GF-AAS model Perkin Elmer AAnalyst 600 followed the standard operating procedures (SOPs) as given by the manufacturer. Other instruments such as turbidity meter, pH meter and weighing scale were operated based on the SOPs of the respective equipment as well. Following SOPs could reduce errors due to analysis. All instruments were calibrated before use. For GF-AAS, a calibration curve of approximately 1 was obtained prior to analysis of water samples so that the absorption of the lead atom became more accurate. Pre-testing of questionnaires was conducted on 10% of sample size before data collection to ensure that every question asked in questionnaires could be easily understood and thus answered by respondents. Pre-testing was conducted on 10 households in Kajang area, approximately 10kms from the study area.

### 3.3 Risk Assessment

The following equation was used to calculate the daily exposure for ingestion route (US EPA, 1999; Chrostowski, 1994).


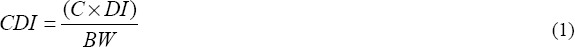


Where, CDI = Chronic daily intake (µgkg^-1^ day^-1^),

C = Lead concentration in water (mgL^-1^),

DI = Average daily intake rate of water (LDay^-1^),

BW = body weight (kg).

Values of these three input variables, unique to each respondent, were used to estimate the respondent's individual chronic daily exposure level. The hazard quotient (HQ) was calculated to estimate non-carcinogenic risk using the following equation:


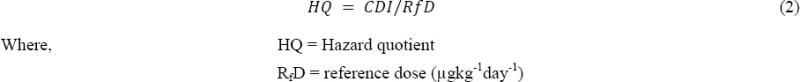


A HQ value of >1 implied a significant risk level. R_f_D value employed in this study was referred to Provisional Weekly Tolerable Intake (PWTI) of 25 μg of lead per kg of body weight (equivalent to 3.5 μgkg^-1^ of body weight per day) established by JECFA for infants and children but extended to all age groups in 1993 (WHO, 2003).

## 4. Results

### 4.1 Sociodemographic Background

The socio demographic data of respondents are summarized in Table 2. Respondents’ age ranged from 18 to 74 years old, with a mean of 41.09± SD 15.22 years old. The largest age group was 20-29 years old (29%). There were 34 Malays, 54 Chinese, 10 Indians and 2 other minority races who participated in the study. The mean body mass indexes (BMIs) of the respondents fell in the healthy and normal range of 25.34± SD 6.56kgm^-2^. In the study, 26% of the respondents were degree holders, 23% of them had completed primary school education level while 4% of them did not receive any formal education. From the questionnaire, it was found that 48% of the respondents had monthly income of less than RM720, 12% of them have monthly income in the range of RM720 to RM1500, 25% of them in the range of RM1501- RM2500 and 15% of them had a higher income of more than RM2500.

**Table 2:** Sociodemographic information (n=100)

**Variable**	**Mean ± S.D**	**Median**	**Min Value**	**Max Value**
Age	41.09± 15.22	40.50	18.00	74.00
Weight (kg)	67.39± 17.63	64.00	42.00	157.00
Height (m)	1.63± 0.08	1.63	1.45	1.78
BMI(kgm^-2^)	25.34± 6.56	24.66	14.88	60.60

**Variable**	**Category**	**Frequency**	**Percentage, %**	**Cumulative Percentage**
Gender	Male	51	51	51
	Female	49	49	100
Races	Malay	34	34	34
	Chinese	54	54	88
	Indian	10	10	98
	Others	2	2	100
Marital Status	Single	35	35	35
	Married	62	62	97
	Divorced	3	3	100
Highest Education Level	No schooling	4	4	4
	Primary school	23	23	27
	SRP/PMR	20	20	47
	SPM	13	13	60
	STPM/Diploma	14	14	74
	Degree	26	26	100
Monthly Income (RM)	< 720	48	48	48
	720 – 1500	12	12	60
	1501 – 2500	25	25	85
	> 2500	15	15	100

### 4.2 Lead Concentrations in Water Samples

The parameters tested in the water samples were lead concentration, temperature, pH, TDS and conductivity. The results are summarized in Table 3. For lead concentrations in the first-flushed samples, the results ranged from 0.097µg/L to 56.490µg/L. The mean lead concentration was 3.041± SD= 6.967µg/L while the median lead concentration was 1.313µg/L. As compared with National Drinking Water Quality Standard (NDWQS), the lead concentration in 4 first-flushed samples had exceeded the acceptable values of 10µg/L. For fully-flushed samples, the lead concentration ranged from 0µg/L to 5.215µg/L. The mean lead concentration was 1.064 ± SD 1.103µg/L with median of 0.702µg/L. None of the samples had surpassed the NDWQS value. Lead was not even found in 2 of the fully-flushed samples.

**Table 3:** Lead concentration and physical properties of water samples and the comparison with available standards

**Parameters**	**Lead concentration in first-flushed water (µg/L)**	**Lead concentration in fully-flushed water (µg/L)**	**Temperature (°C)**	**pH**	**Conductivity (µS/cm)**	**Total Dissolved Solids, TDS (mg/L)**
**Mean**	3.041	1.064	26.86	8.19	25.75	12.87
**Median**	1.313	0.702	27.35	8.08	26.30	13.15
**Std Deviation**	6.967	1.103	2.44	0.48	2.31	1.15
**5^th^ Percentile**	0.169	0.034	20.91	7.66	21.41	10.71
**10^th^ Percentile**	0.479	0.079	23.13	7.76	22.04	11.22
**25^th^ Percentile**	0.675	0.370	26.23	7.91	24.2	12.1
**Median**	1.313	0.702	27.35	8.08	26.3	13.15
**75^th^ Percentile**	2.546	1.445	28.3	8.31	27.3	13.68
**90^th^ Percentile**	5.297	2.473	29.09	8.73	28.3	14.1
**95^th^ Percentile**	8.133	4.057	30.47	9.58	28.7	14.3
**Min value**	0.097	0.000	19.2	7.16	16.88	8.4
**Max value**	56.490	5.215	31.6	9.79	30.60	15.3
**Drinking water standards**	10^a^	10^a^	Not available	6.5-9^a^	500^b^	1000^a^

a National Drinking Water Quality Standard 2009

b Water Supply (Water Quality) Regulations 1989

For other physical properties, none of the parameters had exceeded the NDWSQ and USEPA limit, except for pH value. There were 8 water samples which exceeded the NDWQS limit of pH 9. However, the mean pH value which was 8.19 and median pH value of 8.08 were, in fact, in the range of acceptable value. Conductivity and TDS of water samples were far below the NDWQS limit. According to Table 4, no significant correlation was found between lead concentration and other physical properties of the water such as pH, TDS, conductivity and temperature (p>0.05).

**Table 4:** Spearman Correlation of lead concentration with other physical properties

**Lead concentration**		**Conductivity (µS/cm)**	**TDS (mg/L)**	**pH**	**Temperature (°C)**
**in first-flushed water (µg/L)**	Correlation Coefficient	0.129	0.131	-0.116	0.167
	Sig. (2-tailed)	0.200	0.195	0.252	0.097
	n	100	100	100	100
					
**fully-flushed water (µg/L)**	Correlation Coefficient	0.175	0.179	0.031	0.117
	Sig. (2-tailed)	0.081	0.074	0.758	0.247
	n	100	100	100	100

The study showed that 78 first-flushed water samples had higher lead concentration than fully-flushed water. As shown in Table 5, there was a significant difference of lead concentration between first-flushed water and fully-flushed water, and first-flushed water has higher lead concentration as compared with fully-flushed water. Flushing had elicited a statistically significant change in lead concentration in the water (Z = -5.880, p <0.05).

**Table 5:** Difference of lead concentration between first-flushed and fully-flushed water

**Pair**	**Ranks**	**n**	**Mean Rank**	**Sum of Ranks**	**Z**	**p**
Lead concentration in fully-flushed water samples –first-flushed water samples	Negative Ranks	78^a^	54.29	4235.00	**-5.880^d^**	**0.01**
	Positive Ranks	22^b^	37.05	815.00		
	Ties	0^c^				
	Total	100				

a Lead concentration in fully-flushed water samples < first-flushed water samples

b Lead concentration in fully-flushed water samples > first-flushed water samples

c Lead concentration in fully-flushed water samples = first-flushed water samples

d Based on positive rank

### 4.3 CDI and HQ Values

As summarized in Table 8, the mean daily drinking water intake rate value found in this study was 1.794L/Day while the mean body weight of the respondents was found to be 67.39kg and median weight was 64kg. The CDI value ranged from 0 to 0.230µgday^-1^kg^-1^, with mean CDI value at 0.028± SD 0.034 µgday^-1^kg^-1^ and median at 0.017µgday^-1^kg^-1^. Mean HQ value was 0.008± SD 0.0097 while the median was 0.0048. None of the respondents had HQ level of more than 1. HQ values were less than 1 in the whole population indicated that the non-carcinogenic risk associated with exposure to lead in tap water via ingestion pathway was negligible.

**Table 8:** Daily intake rate of water, body weight and chronic daily intake, CDI

**Parameters**	**Daily intake rate of water (L/day)**	**weight (kg)**	**CDI (µgday^-1^kg^-1^)**	**HQ**
Mean	1.794	67.39	.028	0.0080
Median	1.600	64.00	.017	0.0048
S.D.	1.144	17.63	.034	0.0097
75^th^ Percentile	2.000	75.00	.030	
90^th^ Percentile	3.000	85.00	.073	0.0208
95^th^ Percentile	4.190	94.80	.096	0.0275
99^th^ Percentile	7.586	156.83	.226	0.0645
Minimum	.400	42.00	.000	0.0000
Maximum	7.600	157.00	.230	0.0647

## 5. Discussion

### 5.1 Lead Concentrations and Physical Properties of Water Samples and the Comparison with Available Water Standards

No similar study assessing the risk of exposure to lead in tap water was found in Malaysia. However, a study from Saraswathy (2009) at the Sunway residential area found that lead concentration in 4 stagnant water samples collected from consumer taps had actually exceeded the NSDWQ value of 10µg/L.

Rajaratnam et al. (2002) found that lead level in first-flushed water in the Sydney metropolitan area was very high, with a mean lead content of 29± SD= 42µg/L. The study found that 60% of the first-flushed water samples collected was higher than the Australian Drinking Water Guidelines (ADWG) limit value of 10µg/L. As compared with this study, the mean lead concentration from the Sydney study was around 10 times higher than in SK in first-flushed water samples. The huge difference of lead concentration may be due to age of houses. The study by Rajaratnam et al. focused only on new houses while this study did not take into consideration the age of house as the inclusive criteria for sampling. Sharrett et al. (1992) found that new housing areas of less than 5 years old or residence in which recent plumbing renovation or repairs had been completed, has the potential of producing higher lead exposure than older housing areas because the combination of copper piping with lead solder produces galvanic corrosion that can leach lead even in relatively non-corrosive water. Soldered connections in recently built homes fitted with copper piping can release enough lead (210–390 μg/l) to cause intoxication in children (Cosgrove et al., 1989).

A study done by Fertmann et al. (2004) in Hamburg, Germany found that less than 25% of the fresh water samples taken after 3 minutes of flushing had exceeded 10µg/L among 248 water samples collected. It showed that lead concentration in flushed sample was low. A similar finding by Kavcar (2009) showed that lead was only detected in 15% of the drinking water samples collected. The concentration of lead was too low that it was undetectable in the rest of the samples.

In this study, the mean pH value which was 8.19 and median pH value of 8.08 were in the range of acceptable value. The result was similar with the study by Zietz et al. (2003) in Lower Saxony, Germany who found that tap water was slightly basic and the mean pH value was 7.83. According to Konsortium Abass Sdn Bhd, which is the operator for the Sungai Semenyih Water Supply Scheme, lime is added to the treated water to adjust the pH to the specific level. It was an alkaline compound that was added to neutralize the effect of alum, which was an acidic salt. It was important to increase the pH value of drinking water because a pH below 7 indicated an acidic water condition which is corrosive to metal pipes. This not only means that the pipes and faucets can be damaged by low pH water, but also that the water can contain high levels of copper, lead, or zinc that have corroded out of the plumbing systems (ATSDR, 2007).

Total dissolve solids (TDS) is the term used to describe the inorganic salts and small amounts of organic matter present in solution in water while conductivity is a measure of the conductance of water to an electric current. The conductivity values indicated the high mineralization of the samples. In this study, mean TDS and conductivity values of the water were far below drinking water standards. Unlike SK, TDS and conductivity value of tap water samples in Haiti were found to be very high and exceeding the limit value (Angerville et al., 2004). Reliable data on possible health effects associated with the ingestion of TDS in drinking water were not available (WHO, 1996).

### 5.2 Correlation of Lead Concentration with other Physical Properties

For pH, the result was consistent with the study done by Kim and Herrera (2011), that significant correlations between lead concentrations and pH were not observed. Le et al. (2003) concluded that there was no correlation between lead in tap water and pH levels as well. However, in a study of drinking water with a low alkalinity and a fairly low pH, high levels of lead were found in the drinking water of households that had lead plumbing (McFarren et al., 1977). In this study, pH was not significantly correlated with lead concentration most probably because the pH level of the water samples were just slightly alkali, with a mean of 8.19, and generally at low pH levels only (<6.8), most metals would corrode more rapidly than at higher pH (>9.0) (Schock & Gardels, 1983).

In this study, temperature was not correlated with lead in tap water. The study was inconsistent with other studies which found that seasonal variations in temperature between the summer and winter months were correlated with lead concentrations, with the warmer temperatures of the summer months increasing lead concentrations (Karalekas et al., 1983; Colling et al., 1987; Douglas et al., 2004). This inconsistency might be due to the temperature of water samples did not vary much as Malaysia was a tropical country with hot weather all year round, as compared with studies done in other countries where extreme temperature changes are common especially during the winter and summer months.

TDS and conductivity were found to be not correlated with lead concentration in water samples. Although there was no correlation between TDS and lead concentration, there were studies suggested that certain components of TDS, such as chlorides, sulphates, magnesium, calcium, and carbonates, affect corrosion or encrustation in water-distribution systems. High TDS levels (>500mg/L) resulted in excessive scaling in water pipes, and one must consider that lead concentration in tap water was contributed mainly by the corrosion of pipes (WHO, 1996).

### 5.3 Comparison of Lead Concentration in First-Flushed and Fully Flushed Samples

The result was consistent with a study done in Sydney, where lead concentrations were significantly different in the three types of samples. Multiple-comparison tests showed that lead levels in first-flushed samples were significantly higher than those in either post first-flushed or fully flushed samples (Rajaratnam et al., 2001).

The finding that first-flushed samples had higher lead concentration than fully-flushed samples was generally in agreement with other literature (Gulson et al., 1994; Schock et al., 1988; Levin, 1986; Sharrett et al., 1982). The difference might be due to standing time of water in the pipes. Several studies had found that first-flushed water had higher lead concentration than fully-flushed water because of the standing time. Schock et al. (1996) concluded that lead levels rapidly increase upon stagnation, but ultimately approach a fairly constant equilibrium value after overnight stagnation. Lytle and Schock (2000) showed that lead levels increased rapidly with the stagnation time of the water, with the most critical period being during the first 20-24 hours.

Since the level of trace metals increased upon stagnation of water, flushing the water present in the plumbing system would significantly reduce the levels of lead and copper. A study by Gardels et al. (1989) showed that 60-75% of the lead leached from common kitchen faucets appears in the first 125 mL of water collected from the faucet. In a study on contamination of tap water by lead solders, Wong and Berrang (1976) concluded that the first 2L of water from cold water taps should not be used for human consumption if the water has been stagnant for a day. In Canadian studies, in which the cold water tap of homes was flushed for 5 minutes, no levels of trace metals exceeded their respective Canadian drinking water guidelines (Méranger et al., 1981; Singh and Mavinic, 1991).

### 5.4 Exposure and Risk Assessment

The exposure assessment in this study was done based on deterministic approach. In this approach, exposure estimated individually for each subject using his own body weight and daily intake rate of water instead of using USEPA daily intake rate of water default value of 2L/day and an adult weight of 70kg. It was to avoid overestimation or underestimation of population risk (Kavcar et al., 2006).

The mean value for SK population was found to be close to the USEPA default value of 2L/day. Many studies had found that the daily intake rate of water were different across different regions and countries due to different climate. Gillies and Paulin (1983) suggested an average daily water intake rate of 1.256L/Day and a 90th percentile rate of 1.9 L/Day on the basis of a survey conducted in New Zealand. A study done in Korea found that the daily intake rate of water was 2.56L/day (Ji et al., 2009).

Daily water intake rate of the SK population was found to be less than USEPA default value of 2L. The reason might be due to the weather. As stated earlier, the data collection was conducted from late December until early February. According to the Malaysian Meteorological Department, December and January are the months with the lowest average monthly temperature. When the weather is cooler, the daily water consumption will become less. It was supported by study of Kavcar et al. (2006) who found that daily intake rate of water varies according to climatic condition.

The values for respondents’ weight were found less than the value suggested by the USEPA (70 kg) and used in many studies (Williams et al., 2002; Lee et al., 2004). If the body weight was assumed to be 70 kg for the SK population, exposure and risk would have been underestimated for female participants and overestimated for male participants. Chronic daily intake (CDI) was calculated for each respondent based on his daily intake rate of water, body weight and the lead concentration found in fully-flushed water.

In order to estimate risk, hazard quotient, HQ was calculated by dividing CDI by the reference dose, R_f_D of 3.5μg/kg of body weight per day as suggested by JECFA. The HQ values were found ranged from 0 to 0.0647. HQ values were less than 1 in the whole population indicated that the non-carcinogenic risk associated with exposure to lead in tap

### 5.5 Study Limitations

There were three main limitations in this study. The first limitation was that only the ingestion route was taken into consideration in order to assess exposure associated with lead in drinking water. The second limitation was recall bias as the daily intake rate of water was determined not based on respondent`s water intake rate in 7 consecutive days, but by asking the respondents to recall back the daily water intake rate using standard cup of 200ml. Besides, there was a big variation of number of water samples collected from each housing area that the lead concentration in the water samples from these areas could not be compared statistically. For example, 48 water samples were collected from *Taman Bukit Serdang* but only 11 water samples were collected from Taman *Serdang Jaya*. It was because the number of water samples collected was determined by the geographical population distribution where the number of water samples collected was a representative of the population distribution in that area.

## 6. Conclusion

The overall quality of water supply in SK was found to be satisfactory because most of the parameters tested in this study were within the range of permissible limit and only a few samples had exceeded the standard values for lead concentration and pH values. The study also found that first-flushed samples had higher lead concentration than fully-flushed samples and flushing would significantly reduce the levels of lead. In conclusion, none of the respondents was found to have HQ level more than 1, indicated that the non-carcinogenic risk of exposure to lead in tap water among residents of SK was negligible. Although the risk is negligible, we have to realize that the maximum permissible levels of elements established by the WHO and national legislations are generally based on toxicological studies on animals. Little is known in relation to the long-term human exposure to harmful elements in water. More research and surveillance studies should be conducted to determine exposure on the Malaysian population with regards to trace metals, especially lead in drinking water and to know the long-term effect of these elements to human health.
